# Olfactory Receptors in Semen and in the Male Tract: From Proteome to Proteins

**DOI:** 10.3389/fendo.2017.00379

**Published:** 2018-01-23

**Authors:** Domenico Milardi, Claudia Colussi, Giuseppe Grande, Federica Vincenzoni, Francesco Pierconti, Francesca Mancini, Silvia Baroni, Massimo Castagnola, Riccardo Marana, Alfredo Pontecorvi

**Affiliations:** ^1^Division of Endocrinology, Fondazione Policlinico Universitario Agostino Gemelli, Rome, Italy; ^2^International Scientific Institute Paul VI, Catholic University, Rome, Italy; ^3^Institute of Cell Biology and Neurobiology, National Research Council, Monterotondo, Italy; ^4^Institute of Chemistry and Clinical Biochemistry, Catholic University, Rome, Italy; ^5^Department of Pathology, Catholic University, Rome, Italy

**Keywords:** olfactory receptors, sperm, spermatogenesis, OR4S1, OR4C13, OR1I

## Abstract

The estimated number of testicular olfactory receptors (ORs) in mammals range between 20 and 66. Previous data reported the role of hOR17-4 and mOR23 in sperm–oocyte chemiotaxis. Proteomic analysis was performed to understand which are the ORs expressed in seminal plasma. Seminal samples by four fertile men were analyzed by an Ultimate 3000Nano/Micro-HPLC apparatus coupled with an LTQ-Orbitrap XL hybrid mass spectrometer. Western blot analysis confirmed the expression of three identified ORs. The expression of ORs in sperm cells, testis, and epididymis was evaluated by confocal microscopy analysis. In seminal plasma eight different ORs were identified by proteomics and three ORs have been confirmed by western blot. Confocal microscopy analysis revealed that OR4S1, OR4C13, and OR1I1 are expressed on the surface of sperm cells. In testicular tissue, OR4S1 and OR1I1 are expressed in spermatocytes and spermatids and OR4C13 is expressed throughout all the tubules. In patients with spermatocyte maturation arrest OR4S1 and OR1I1 expression was reduced and a weak positivity for OR4C13 was detected in the spermatogonia. OR4S1, OR4C13, and OR1I1 had intense and diffuse staining in the epididymis. This study initiated a new methodology for screening OR repertoire in sperms, testis and epididymis. Our results open new insights into OR involvement in sperm maturation and migration.

## Introduction

Olfactory receptor proteins (ORs) are G-protein-coupled receptors which represents the largest gene family in the human genome. About 1,000 ORs are encoded by the genome (1). ORs are expressed in the nasal olfactory sensory neurons, which are involved in pathways of signal transduction. The signal transduction leads to the stimulation of membrane adenylate cyclase III and at least to the increase of intracellular Ca^2+^ and Na^+^ concentrations. These events induce membrane depolarization (2). ORs are also involved in chemotaxis and cytokinesis by exerting a regulatory role on the cytoskeleton (3).

Some of these receptors are expressed in non-olfactory tissues, including myocardial and erythroid cells (4, 5), ganglia of the autonomic nervous system (6), spleen (7), colon (8), prostate (9) and also in testis (10).

The physiological function of the testicular ORs was debated, but it remains still unknown.

The estimated number of testicular ORs in mammals ranges between 20 and 66. Twenty mammalian OR genes have been identified, in fact, having a predominant expression in the male germ line (11), most notably during late stages of spermatogenesis. Parmentier’s group, using RNase protection assays, reported that an overall of 50 receptors were expressed in mammalian testis (12). Similar numbers of ORs were subsequently confirmed in mouse testis, using a high-throughput oligonucleotide microarray approach, detecting 66 OR enriched genes (13). Specific ORs have been localized at the midpiece and base of the flagellum of mature sperm (14, 15).

A specific testicular OR, the hOR17-4, was identified by RT-PCR in biopsies of human testicular tissue (16). Its surface expression was subsequently confirmed on mature sperm by proteomic analysis using a Multi-dimensional Protein Identification Technology (14).

The specific expression profile of the different ORs in testis tubule, as well as their function, is still not clear. Previous data have been reported suggesting a role for hOR17-4 and mOR23 in sperm–oocyte chemiotaxis. In 2003, Spehr et al. demonstrated that hOR17-4 might represent a mechanism for the sperm cells to detect chemotactic stimuli and to translate this information into stereotyped motion patterns (16).

Aside from the chemotactic role of hOR17-4 and mOR23, other testicular ORs might be involved in spermatogenesis, epididymis maturation and sperm function.

In a previous study, we experimented for the first time a proteomic approach for the study of the seminal plasma in fertile males (17).

In this context, the identification of seminal OR repertoire is an interesting field in the research of reproduction. It was the aim of the present study to apply an high-resolution mass spectrometry (MS) based shotgun proteomic approach in order to define the repertoire of seminal ORs and to verify the expression of newly identified ORs in sperm cells and testicular and epididymal tissue.

## Materials and Methods

The protocol was approved by the Ethical Committee of Fondazione Policlinico “A. Gemelli”, Rome (Italy). All subjects gave written informed consent in accordance with the Declaration of Helsinki.

### Human Subjects

Eight fertile men, whose partners were pregnant when the study was started, participated in this study. None had a history of fertility problems. All female partners conceived within 3 months before the start of the study.

Exclusion criteria from the study were: men who younger than 20 or older than 55 years, testicular diseases, and presence of varicocele and/or genital tract infections.

The subjects gave informed consent according to the guidelines of the Declaration of Helsinki.

### Hormonal Study

A blood sample was collected at 08:00 a.m. for the determination of LH, FSH, testosterone (T), estradiol (E2), dihydrotestosterone (DHT), sex hormone-binding globulin (SHBG), and prolactin (PRL) levels. LH, FSH, and SHBG were assayed by immunoradiometric methods on a solid-phase, with the use of a monoclonal double-antibody technique. T, E2, and PRL were assayed in duplicate by RIA with the use of commercial kits (Radim). DHT was assayed by RIA with the use of a commercial kit (Chematil).

### Semen Analysis and In-Solution Digestion

Semen samples were obtained in all subjects after 3 days of sexual abstinence. Volume, pH, sperm concentration, motility, and normal morphology were determined according to World Health Organization (2010) guidelines for semen analysis, after liquefaction at room temperature.

### Proteomic Analysis

Liquefied semen samples were centrifuged at 9,200 *g* for 20 min to obtain the seminal plasma and to ensure complete removal of the cellular components. After the centrifugation, an aliquot was checked to confirm that no spermatozoon was present. Seminal plasma was divided in 0.5-mL aliquots, which were immediately frozen at −80°C until MS analysis was carried out (within 1 month).

An aliquot of each seminal plasma corresponding to 1 mg of total protein (as measured by a Bradford assay) was subjected to an in-solution digestion protocol as described previously (17). For proteomic analysis, the samples were resuspended in aqueous trifluoroacetic acid [TFA/H_2_O 0.2% (v/v)] and equal protein quantity (5 µg) of each sample was analyzed. HPLC–MS analyses were performed by Nano/Micro-HPLC apparatus (Ultimate 3000, Dionex) coupled to LTQ-Orbitrap XL hybrid mass spectrometer (ThermoFisher). The samples were resuspended in aqueous trifluoroacetic acid solution (TFA/H_2_O 0.2% v/v) and an equal protein quantity (5 µg) was analyzed onto C18 column (3 µm particle diameter; 1 mm inner diameter × 10 cm; Supelco Discovery Bio Wide Pore), using TFA/H_2_O (0.056%, v/v) and TFA (0.05%, v/v) in acetonitrile/water solution (80:20, v/v) as eluents A and B, respectively. The linear gradient was performed from 0 to 50% of solvent B in 60 min at flow rate of 80 µL/min. High-resolution MS and MS/MS spectra were recorded in data-dependent scan mode, where every three MS the spectrometer performed one full mass spectrometric scan (60,000 resolving power) and three MS/MS scans on the most intense multiple-charged ions selected, fragmented by collision-induced dissociation (35% normalized collision energy).

### Data Analysis

The HPLC–MS and MS–MS analysis were carried out simultaneously.

The MS data were elaborated by Proteome Discoverer software (version 1.3 Thermo Fisher Scientific) against UniProtKb/Swiss-Prot protein knowledge database (release 2017_02: *Homo sapiens*). The setting parameters were the following: trypsin enzyme with three missed cleavages, carbamidomethylation of cysteines as fixed modification, and oxidation of methionines, phosphorylation of threonines, tyrosines, and serines as variable modification.

To obtain a reliable identification of the peptides, the following stringent filters were used: false discovery rate 1%, almost two peptides per proteins.

To define the repertoire of ORs involved in seminal plasma, we considered the list of all the identified ORs among the merged data of identified proteins in the samples.

The label-free quantification of proteins was performed *via* Precursor Ions Area Detector Node during the bioinformatic analysis using Proteome Discoverer software. This quantification method was used to obtain an idea of the relative quantities of all peptides in a sample. The Proteome Discoverer application calculates peptide areas during processing, using them to automatically calculate protein areas for the proteins in the report. It calculates the area of any given protein as the average of the three most abundant distinct peptides identified in the protein. Results are reported as average ± SD.

### Western Blot

Western blot analysis of seminal plasma were performed treating samples with Reducing Laemmli buffer (31.5 mM Tris–HCl pH 6.8, 1% SDS, 10% glycerol, 0.005% bromophenol blue with 355 mM 2-mercaptoethanol) and boiled at 95°C for 5 min. Proteins were separated by SDS-PAGE and were transferred onto PVDF membrane from Millipore (Milan, Italy). Primary antibodies used: rabbit polyclonal anti-OR4S1, anti-OR1I1, and anti-OR4C13 antibodies (Abcam 1:500), mouse monoclonal anti-actin (Sigma). Membranes were developed using the enhanced chemiluminescence (ECL Amersham) by chemiluminescence imaging system, Alliance 2.7 (UVITEC Cambridge) and quantified by the software Alliance V_1607.

### Testicular and Epididymal Samples

Formalin-fixed and paraffin-embedded testicular and epididymal tissues belonging to five patients submitted to emasculation surgery for penile cancer with scrotal erosion (median age 37 years) in the University Hospital “Policlinico Universitario A. Gemelli” from 2004 to 2011 were analyzed to evaluate the testicular expression of OR4S1, OR4C13, and OR1I1. The examination revealed no invasion of the testis by the penile cancer.

In addition, formalin-fixed and paraffin-embedded testicular tissues, coming from testicular biopsies of five patients with secretive azoospermia and spermatocyte maturation arrest, were analyzed. Hormonal levels of LH, FSH, T, E2, DHT, SHBG, and PRL were retrospectively evaluated.

Sections of a testicular biopsy, finding a “Sertoli cell only syndrome,” were used as negative control.

### Confocal Microscopy Analysis on Sperm Cells, Testis and Epididymis

Spermatozoa were mounted on coverslips and fixed in 2% paraformaldehyde for 20 min at room temperature and then permeabilized in 0.1 M phosphate buffered saline (PBS) with 1% Triton X-100 for 20 min at room temperature.

Tissue sections obtained from testis and epididymis were fixed in 2% paraformaldehyde for 20 min at room temperature and embedded in paraffin. Sections were deparaffinized, then they were subjected to antigen retrival and then blocked with 5% BSA for 1 h.

The slides were incubated with anti-OR4S1, anti-OR1I1, and anti-OR4C13 antibodies (Abcam 1:100) overnight at 4°C. Negative controls were generated by incubating the slide, only with the secondary antibody. After three washes in PBS, they were incubated with FITC-conjugated secondary antibody. Spermatozoa were moreover incubated overnight with Lectin-PNA Alexa Fluor 594-Conjugate to stain the acrosomes (Invitrogen, 1:100). Images were acquired with a confocal laser scanning system (TCS-SP2, Leica Microsystems) equipped with an Ar/ArKr laser for 488-nm excitation and HeNe laser for 543-nm excitation. Nuclei were counterstained with DAPI or TOPRO3.

## Results

In the studied population of eight fertile subjects, the measurement of hormonal values revealed levels within normal limits, as follows (mean ± SD): LH 4.2 ± 2.8 mUI/mL; FSH, 3.9 ± 1.5 mUI/mL, T 4.95 ± 1.2 ng/mL; E2 28.1 ± 8.9 pg/mL; DHT 0.33 ± 0.1 ng/mL; SHBG 35.8 ± 6.3 nmol/L, and PRL 11.2 ± 3.7 ng/mL.

Seminal parameters were within the WHO reference values (2010), as follows (mean ± SD): volume 3.5 ± 1.5 mL; pH 7.4 ± 0.6; sperm number 67 ± 33 million/mL; sperm motility 57.4 ± 10.4%; and normal morphology 17.2 ± 7.3%.

Eight different ORs have been identified in seminal plasma by proteomic analysis, using filtering criteria and merge analysis, as reported in Table 1. For each protein, we reported the following information: UniProt, gene name, molecular weight, isoelectric-point and description. The quantitative proteomic data, reporting the mean protein abundance (×10^6^) ± SD for the identified ORs, is reported in Figure S1 in Supplementary Material.

**Table 1:** Identified olfactory receptors (ORs) in seminal plasma by proteomics.

UniProt	Gene name	MW [kDa]	calc. pI	Description
Q8NGB4	OR4S1	34.78	8.7	OR 4S1
Q8NGP0	OR4C13	34.56	8.35	OR 4C13
Q8NH85	OR5R1	36.68	8.28	OR 5R1
O60431	OR1I1	39.3	7.45	OR 1I1
Q8NG81	OR2M7	34.9	7.58	OR 2M7
Q6IF00	OR2T2	36.2	8.73	OR 2T2
Q8NGZ0	OR4AJ1	37.0	8.85	OR 2AJ1
Q8NG83	OR2M3	34.8	8.13	OR 2M3

**Figure 1:**
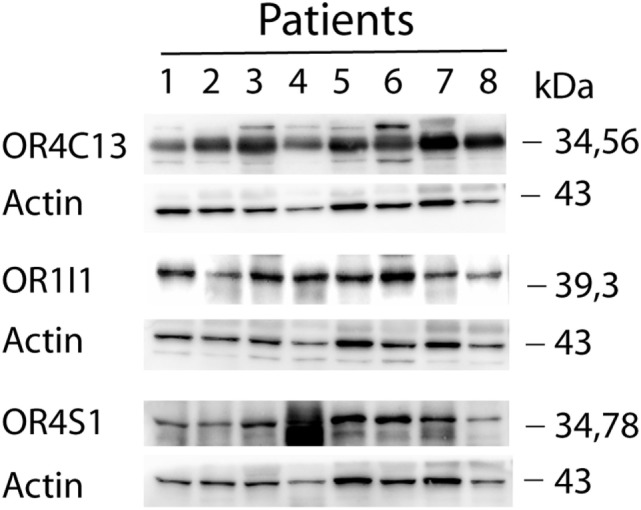
Western blot analysis of OR4S1, OR4C13, and OR1I1 protein levels in seminal plasma from fertile individuals.

Western blot analysis confirmed the expression of OR4S1, OR4C13, and OR1I1 in seminal plasma (Figure 1).

**Figure 2:**
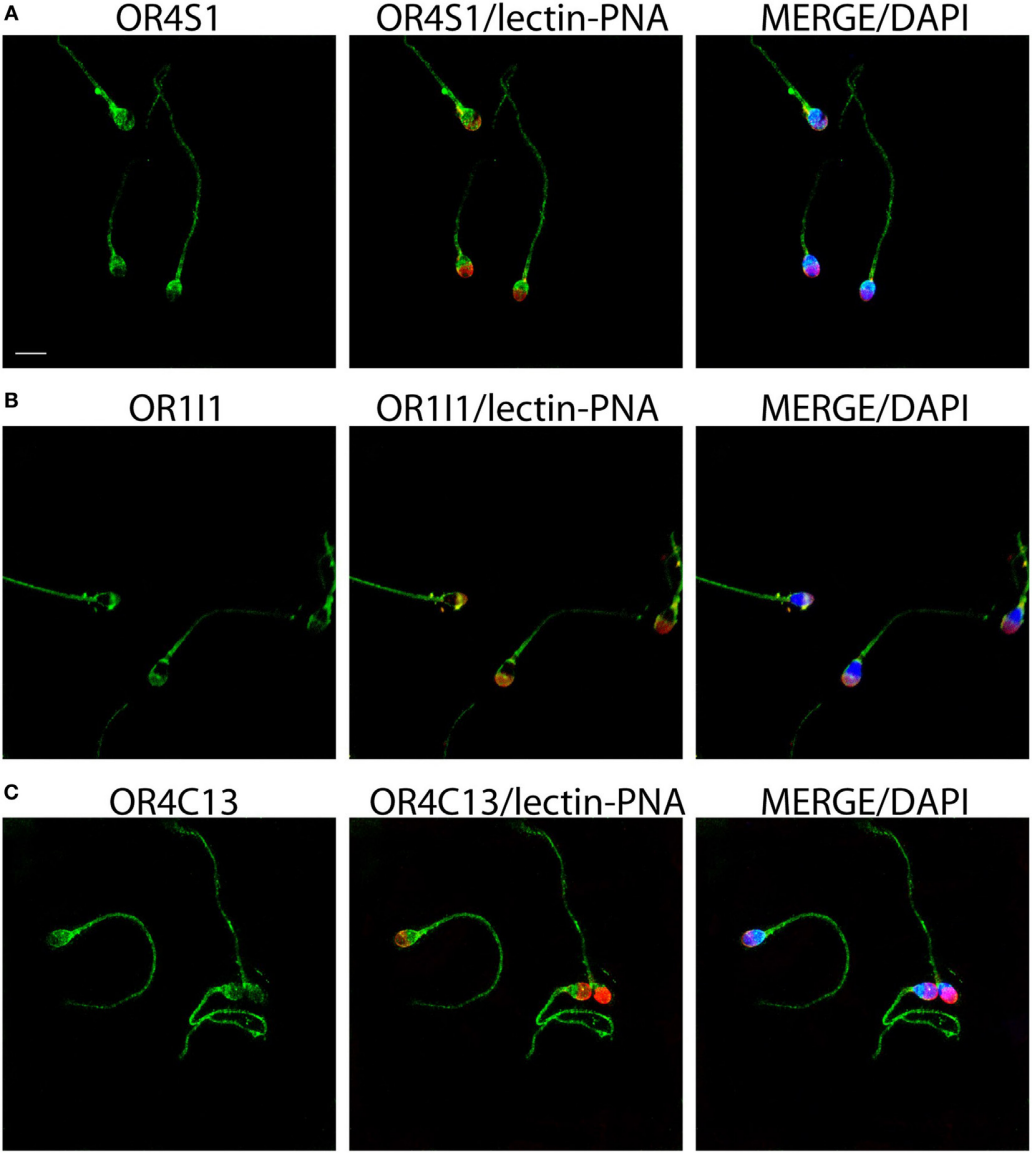
Representative images of OR4S1 **(A)**, OR1I1 **(B)**, and OR4C13 **(C)** protein expression (green) by confocal analysis in sperm cells. Nuclei were counterstained with DAPI and acrosomes with lectin-PNA (red). Scale bar 10 µm.

To confirm whether the expression of these ORs was also common to the sperm cell, we investigated by immunofluorescence the ORs expression of OR4S1, OR4C13, and OR1I1 on the spermatozoa. OR4S1, OR4C13, and OR1I1 were detected on the surface of the sperm cells, including the acrosome, midpiece, and flagellum (Figure 2).

**Figure 3:**
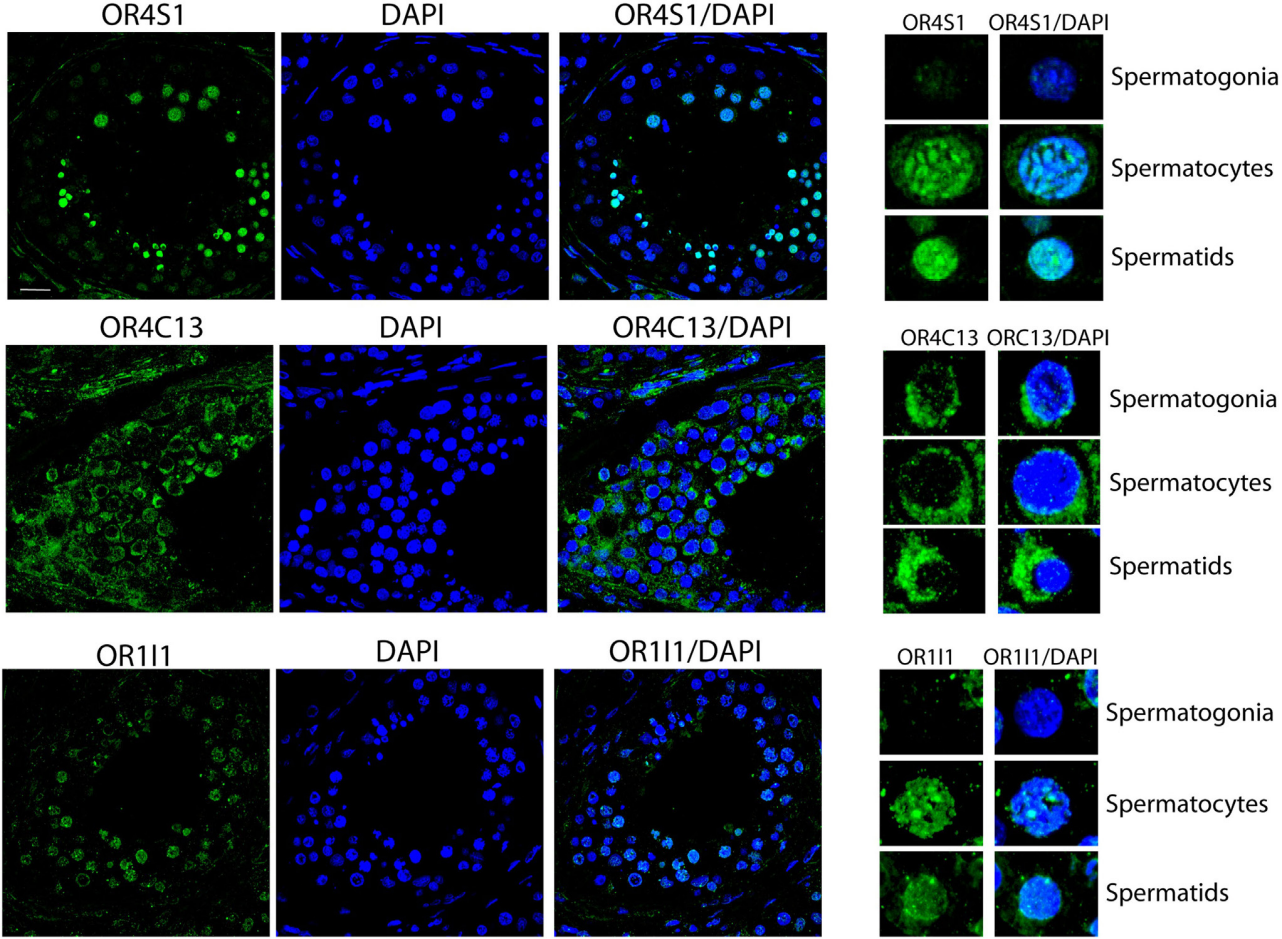
Confocal analysis of OR4S1, OR4C13, and OR1I1 protein expression in testicular tissue. Nuclei were counterstained with DAPI (scale bar 25 µm). Representative panels showing spermatogonia, spermatocytes, and spermatids at high magnification are included in the figure.

Confocal analysis on testicular sections revealed that OR4S1 and OR1I1 expression was mainly localized in spermatocytes and spermatids, but not in the spermatogonia, localized at the basal tubular membrane. OR4C13 immunoreactivity was present throughout the tubules starting from the immature germinal cells (spermatogonia, spermatocytes), to the more differentiated cells (spermatids) (Figure 3).

We, moreover, examined specimens obtained from patients with secretive azoospermia and spermatocyte maturation arrest. In this population of infertile patients, hormonal values were reported as follows (mean ± SD): LH 4.2 ± 3.6 mUI/mL; FSH 7.9 ± 3.2 mUI/mL; T 4.6 ± 1.5 ng/mL; E2 27.2 ± 5.4 pg/mL; DHT 0.21 ± 0.2 ng/mL; SHBG 35.7 ± 5.8 nmol/L, PRL 11.5 ± 3.4 ng/ml.

Statistical analysis confirmed that azoospermic patients with spermatocyte maturative arrest had higher FHS as compared with fertile subject (7.9 ± 3.2 vs 3.9 ± 1.5 mUI/mL, *p* < 0.05), as expected.

**Figure 4:**
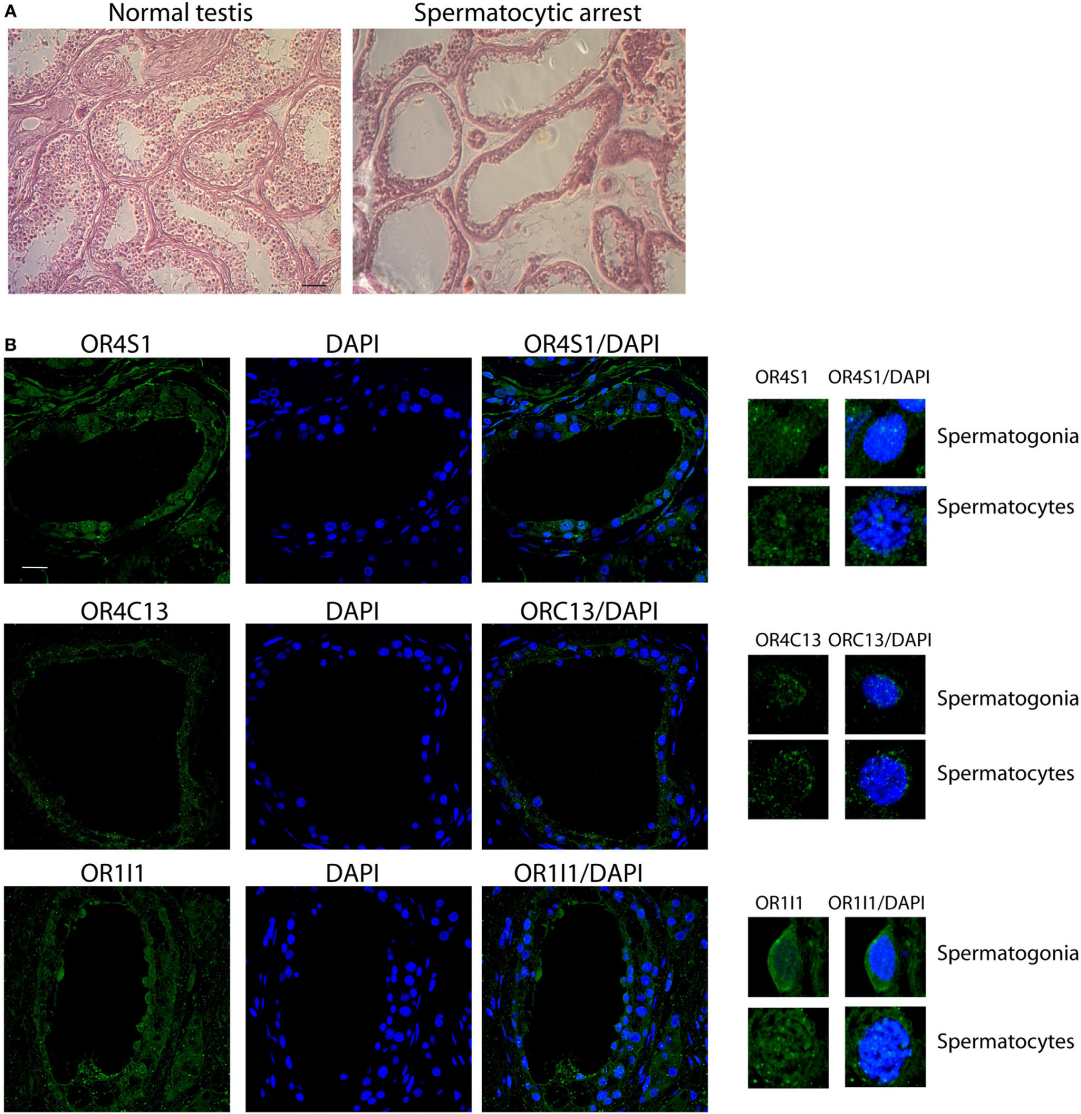
**(A)** Representative images (H&E) of normal or pathological testicular tissue (spermatocyte maturative arrest), scale bar 200 µm. **(B)** Confocal analysis of OR4S1, OR4C13, and OR1I1 protein expression in testicular tissue from patients with secretive azoospermia and findings of spermatocyte maturative arrest. Nuclei were counterstained with DAPI (scale bar 25 µm). Representative panels showing spermatogonia and spermatocytes at high magnification are included in the figure.

In these azoospermic patients, we observed that OR4S1 and OR1I1 expression was reduced in spermatogonia and spermatocytes whereas a weak positivity for OR4C13 was detected in the spermatogonia (Figure 4).

**Figure 5:**
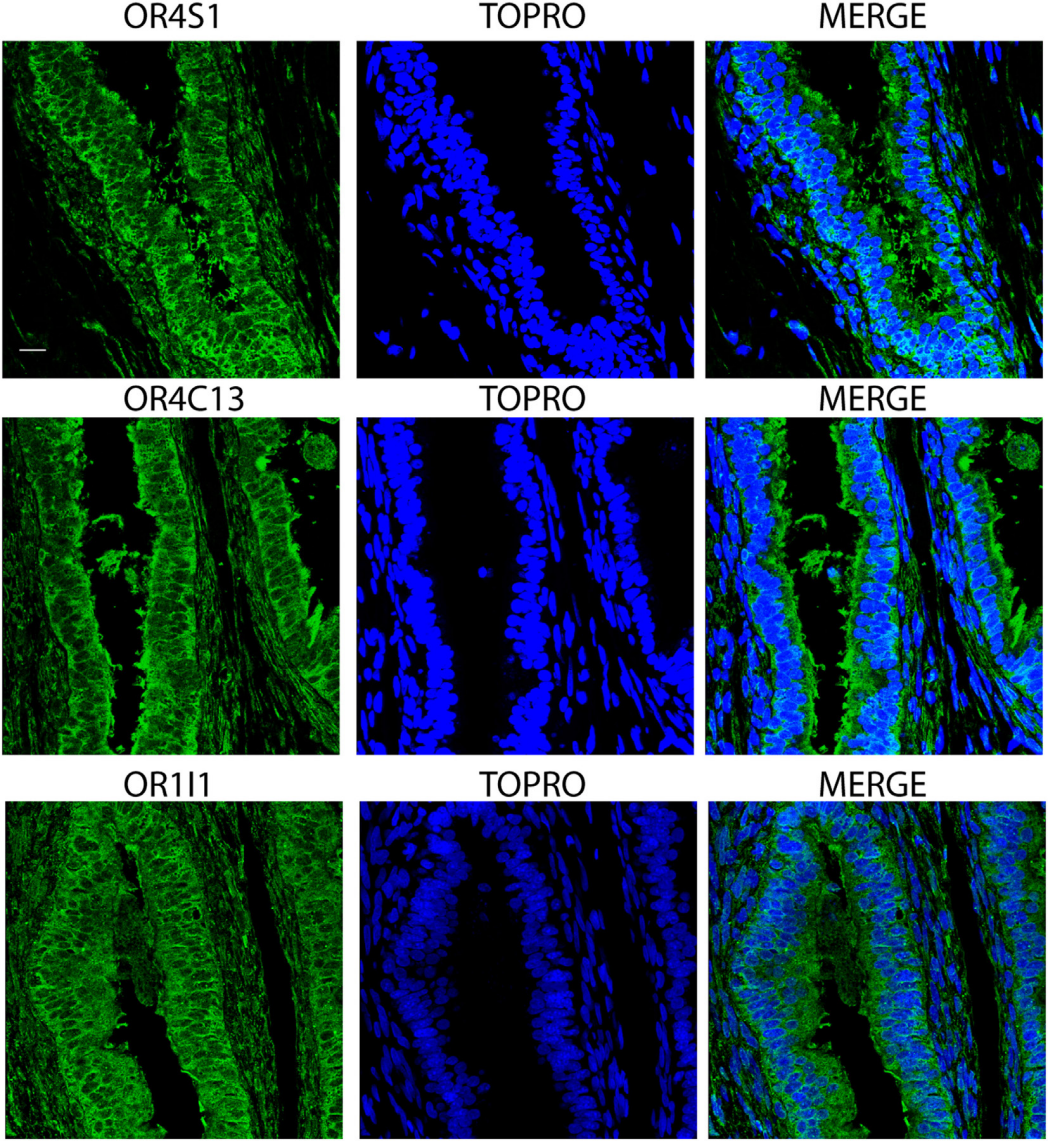
Confocal analysis of OR4S1, OR4C13, and OR1I1 expression in the pseudostratified columnar epithelium of the epididymis. Nuclei were counterstained with TOPRO. Scale bar 25 µm.

Finally, we observed an intense and diffuse staining of OR4S1, OR4C13, and OR1I1 in the pseudostratified columnar epithelium of the epididymis (Figure 5).

## Discussion

The identification of seminal OR repertoire is an interesting field in the research of reproduction. However, a comprehensive human OR catalog in the male germinal tract is still missing.

We developed a new approach to identify human ORs *in vivo* (seminal plasma) comprehensively, applying a high-resolution mass spectrometer approach, confirming the results obtained by this procedure by western blot.

In seminal plasma, we identified for the first time by a proteomic approach the presence of eight novel different ORs. To our knowledge, this is the first wide identification of the repertoire of soluble ORs in seminal plasma. Western blot validated the proteomic data on seminal plasma samples and confirmed the expression of OR4S1, OR4C13, and OR1I1 in seminal plasma.

To confirm whether the expression of these ORs was also common to sperm cell, we investigated by immunofluorescence the ORs expression of OR4S1, OR4C13, and OR1I1 on the spermatozoa. OR4S1, OR4C13, and OR1I1 were detected on the surface of the sperm cells, mainly in the acrosome, the midpiece and the flagellum. Furthermore, another kind of OR receptor, previously studied by Spehr et al., the OR17-4, presents a pattern of localization similar to OR4S1, OR4C13, and OR1I1. Previous studies demonstrated that OR17-4 participates in motility activation and chemiotaxis (14, 16). In recent years, it has become clear that mammalian spermatozoa must be guided in order to reach the oocyte (18). Several studies showed that the mechanism of mammalian spermatozoa-guidance is chemiotaxis (19). In order to explain the sperm–oocyte chemiotaxis, different mechanisms have been proposed, including the interaction between odorants and ORs, both in humans and in animals (20). Ottaviano et al. confirmed the important role of OR17-4 in sperm activity and the putative involvement in idiopathic infertility (21, 22). Like OR17-4, the function of OR4S1, OR4C13, and OR1I1 might be related to acrosome reaction and flagellar motility activation in sperm cells. Further, functional characterizations may provide a clue to understand the roles of OR4S1, OR4C13, and OR1I1 in sperm–oocyte chemiotaxis.

In order to establish the testicular origin of the identified ORs and to investigate whether ORs expression is dependent on the developmental stage in seminiferous tubules, we performed confocal analysis on testicular sections. The specific localization of ORs in different cellular compartments in the tubules should be used as a marker for different cellular populations in the seminiferous tubule.

The main function of OR proteins is olfaction within the chemosensory organ. At the same time, literature data proposed additional OR functions, namely being involved in sperm–oocyte chemotaxis. One of the important questions to address regards which of the hundreds of ORs encoded by the testis is involved in sperm chemotaxis, since such a specific function is unlikely to be mediated by the entire OR repertoire. Previous studies demonstrated that two OR genes, the hOR17-4 (16) and the mOR23 (15), are involved in this function. However, those genes are expressed at low level in testis. Conversely, other OR genes are highly expressed in testis but have not been invoked so far as involved in chemotaxis (23).

Proteomic platforms helped us in exploring the ORs repertoire in seminal plasma providing a spectrum of ORs seminal proteins. In this study, the approach “from proteome to protein” permitted us to verify the expression of three new ORs in sperm cells and to study the different expression pattern in testicular tubules. We observed different tubular localization for the three ORs, we examined. Therefore, we hypothesized that, beside of the sperm chemotactic role, ORs might have specific roles in sperm maturation and migration from basal membrane to the lumen.

Non-chemotactic roles of the ORs have been proposed in literature in numerous other tissues, mostly based on the mere presence of OR transcripts. This includes cell–cell recognition (24) and chemical detection of exogenous or endogenous ligands (25).

Furthermore, we may speculate that the reduction of the expression of the three ORs in azoospermic patients with spermatocyte maturative arrest might represent a cause of the arrest or might represent a consequence of the differences in hormonal (FSH) or genetic pathways. The reduction in the expression of the studied ORs in these patients, although needing confirmation by further studies, might represent a marker to be evaluated in spermatocyte maturative arrest.

Finally, we observed an intense and diffuse staining of OR4S1, OR4C13, and OR1I1 in the pseudostratified columnar epithelium of the epididymis, indicating their function in sperm migration.

In 1993, Vanderhaeghen et al. identified the expression of olfactory transcripts in dog epididymis. In the present study, we report the first identification of the expression of ORs in human epididymis (26). Further studies should help in better understanding the physiology of ORs in the epididymis.

This is the first identification, from a proteomic approach, of the wide OR repertoire in seminal plasma. By the use of a “protein-target” approach, we furthermore demonstrated the presence of OR4S1, OR1I1, and OR4C13 in sperm and in the ducts of testis and epididymis. The presence of these receptors on mature cells might be related to acrosome activity and sperm motility, while their presence in the in testicular tubules and in epididymis might suggest specific roles for these receptors in the regulation of sperm maturation and migration.

In conclusion, despite the limitations of this study (performed on a relatively small sample scale and based solely on descriptive data, not supported by functional analysis for the identified ORs), this is the first application of high-resolution MS-based proteomics at evaluating an array of ORs in seminal plasma and in describing their expression in sperm, testis, and epididymis.

## Ethics Statement

The protocol was approved by the Ethical Committee of Fondazione Policlinico “A. Gemelli.” All subjects gave written informed consent in accordance with the Declaration of Helsinki.

## Author Contributions

DM designed the study and wrote the manuscript; CC performed western blot analyses and confocal microscopy analysis; GG analyzed the data and wrote the paper; FV performed seminal proteomic analysis; FP performed confocal microscopy analysis; FM performed seminal proteomic and western blot analyses; SB performed semen analysis and revised critically the work for substantial contributions; MC, RM, and AP revised critically the work for substantial contributions.

## Conflict of Interest Statement

The authors declare that the research was conducted in the absence of any commercial or financial relationships that could be construed as a potential conflict of interest.

## Supplementary Material

Figure S1 Protein abundance of the identified ORs (×10^6^).

Figure S2 Row data of western blot analysis.
