# Supplementary figures and images for: Olfactory Receptors in Semen and in the Male Tract: From Proteome to Proteins
Source: Front Endocrinol (Lausanne). 2018 Jan 23;8:379. doi: 10.3389/fendo.2017.00379 (PMC5787142; doi:10.3389/fendo.2017.00379)

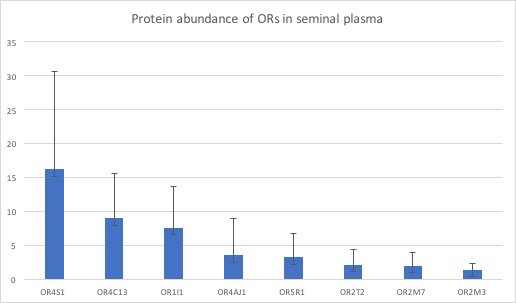

Supplement: Figure S1 — Protein abundance of the identified ORs (×106). [file image_1.tif]

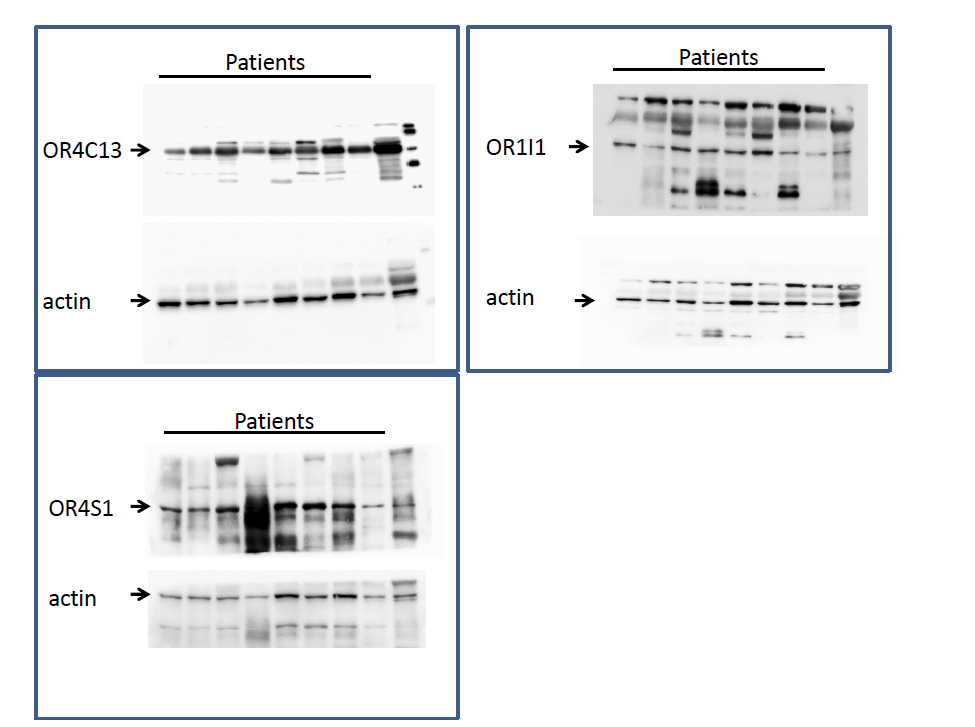

Supplement: Figure S2 — Row data of western blot analysis. [file image_2.tif]
